# The Patient in Precision Medicine: A Systematic Review Examining Evaluations of Patient-Facing Materials

**DOI:** 10.1155/2018/9541621

**Published:** 2018-09-03

**Authors:** Rachel M. Wynn, Katharine T. Adams, Rebecca L. Kowalski, Winnie G. Shivega, Raj M. Ratwani, Kristen E. Miller

**Affiliations:** ^1^National Center for Human Factors in Healthcare, MedStar Health, Washington, DC 20008, USA; ^2^Georgetown University School of Medicine, Washington, DC 20057, USA

## Abstract

Precision medicine (PM) has the potential to tailor healthcare to the individual patient by using their genetic information to guide treatment choices. However, this process is complex and difficult to understand for patients and providers alike. With a recent push in the healthcare community to understand the patient experience and engage patients in their care, it is important to give patients the opportunity to learn about PM. We performed a systematic review to identify previous work assessing the quality of patient-facing PM materials from 2008 to July 2018. Ten studies were identified, which used varying methods and measures. A qualitative assessment was conducted to compare key elements of the studies, including study design, characteristics of the participant population, what measurements were used to assess the PM materials, understandability, preference, psychological reactions, and the type of PM materials being assessed. The studies identified provide important groundwork by highlighting consistent aspects of design that aid in comprehension. Eight of the ten studies focused on the content and organization of genomic test results, while the remaining two assessed educational tools. Two main design elements that appeared across the studies were appropriately designed visual aids and simplified language. The studies identified were limited by the participant populations that were used, which were primarily white and well educated. Only one study attempted to oversample patient populations typically underrepresented in this type of research. Through our systematic review, it is evident that the breadth of knowledge in this field is limited in scope and that more work must be done to ensure that patients can engage in their care when faced with PM.

## 1. Introduction

The State of the Union address delivered by President Barack Obama in January 2015 effectively launched the precision medicine (PM) initiative, raising the awareness of PM's possibilities for many providers and researchers. This initiative encouraged research to advance pharmacogenomics, to identify new targets for treatment and disease prevention, and to lay the scientific foundation for PM to be applied to many different diseases. Since then, numerous disciplines, from oncology to bioinformatics, have released perspective pieces describing PM as the future of medicine and the various obstacles facing it [1–4]. A common theme across these articles is the complexity of PM, which manifests as a barrier to successful implementation for providers and to optimal patient engagement.

PM uses genomic sequencing to identify biomarkers that can be used to understand the nature of one's disease. Biomarkers can be prognostic, providing information about overall outcomes, or predictive, providing information about the likelihood of response to treatment to help optimize therapy decisions. For example, using PM can help oncologists select the most effective treatment and avoid ineffective treatments with harmful side effects [1]. In primary care, PM could be used to inform decisions regarding treatments, such as smoking cessation by examining a patient's speed of nicotine metabolization [5], or inform decisions regarding drinking reduction of alcohol abusers by identifying patients who would respond well to topiramate, a drug that can be used to aid in alcohol abstinence [6].

PM progression and integration into routine clinical care requires buy-in from the patients who also need to understand the associated risks and benefits [7]. Understanding PM is a challenge for patients, as evidenced in multiple studies [8–11]. In one survey, understanding of and attitudes towards genomic testing were assessed for patients with advanced cancer. Results suggest that patients did not understand the distinction between germline sequencing (which examines the genetic code of the patient) and somatic sequencing (which examines the genetic mutations of the tumor). Another survey replicated this finding, demonstrating that oncology patients who are facing decisions about PM are likely to misunderstand the distinction between somatic sequencing and germline sequencing. This confusion reduced their likelihood to participate in somatic sequencing because of concerns regarding information about their genetic code [10]. In order to support fully informed decisions, patients must have an understanding of PM, but research suggests that decisions regarding genetics are driven more by misunderstanding and anxiety than by understanding and values [12]. Additionally, barriers to understanding PM results and education material are unnecessarily complicated language or inaccurate translation, drive misinformation, and decreased participation in testing, especially among patient populations of different socioeconomic status and racial or ethnic minority groups [13]. Patients need to be given the opportunity to learn about genetic concepts and PM in a way that accommodates varying levels of health literacy and engagement and mediates other potential barriers for understanding.

One of the main sources of information for patients is their provider; research shows that PM is unfamiliar and that patients are unlikely to seek information on their own [11]. Unfortunately, clinicians are ill prepared to provide patients with the type of information that is needed to understand PM, as clinicians with the genetic expertise to provide patients with a robust understanding of PM are limited. With increased frequency of PM, the demand for these clinicians is greater than the current supply [14, 15], meaning that there is an increased likelihood that clinicians with limited understanding of genetics concepts will be required to educate patients about them. Research demonstrates that patients are likely to misinterpret risk levels associated with genetic findings and that patients are likely to overestimate the usefulness of PM findings [16, 17].

Due to the complexity of PM, it is important to verify that patient-facing materials are effectively communicating the necessary information. As such, the objective of this review is to identify and report on studies that test the effectiveness of patient-facing PM materials, including educational materials and PM testing results. In response to growing interest from the healthcare community to better understand and improve patient experience, this research identifies components of PM patient-facing materials that improve patient understanding of these complex concepts.

## 2. Materials and Methods

### 2.1. Search Methods

**Figure 1:**
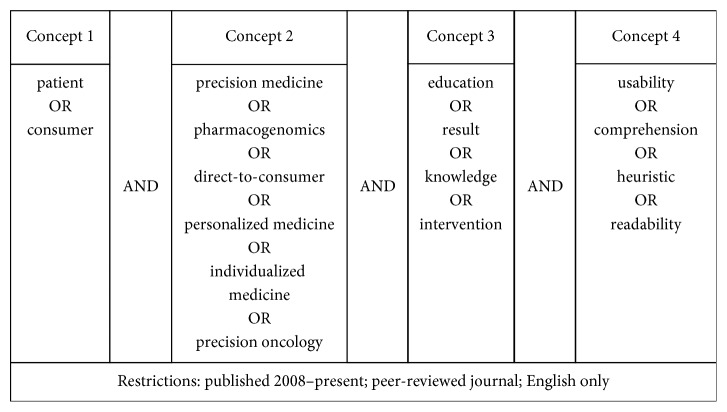
Search terms used in PubMed, Web of Science, and ScienceDirect.

We conducted a systematic review of the literature following the Preferred Reporting Items for Systematic Reviews and Meta-Analyses (PRISMA) checklist. We searched PubMed, Web of Science, and ScienceDirect databases in July 2018 using a combination of search terms to identify articles that reported on the results of empirical studies related to patient or consumer interactions with PM (Figure 1). We focused on the last ten years, as it is only recently that PM has started to be used more widely.

The unique article titles and abstracts were screened by three reviewers (KA, RK, and WS) to determine eligibility for a full-text review. Articles were included if the title and abstract described patient interaction or communication of test results. The full-text of the remaining articles was retrieved and reviewed to determine final inclusion and to capture the details of the study, including methodology, participant population, outcome variables, statistically significant covariates as reported in the study, and the source of the genetic report. Articles were excluded if they were not related specifically to PM or pharmacogenomics (*n*=27), did not focus on genetic test result reports (*n*=26), did not include empirical data (*n*=20), reported repeat results from an included study (*n*=1), or whose primary focus differed from the scope of the review, such as the translation accuracy of genetic test results (*n*=1) or the feasibility of integrating test results into the electronic health record (*n*=1). The references listed in the full-text articles were reviewed to ensure comprehensiveness of the literature review. Articles were only included if they tested the usability, comprehension, or readability of educational materials or results that were specifically related to genomics. This constraint meant that educational materials tested as behavioral interventions were not included nor were studies assessing educational materials regarding general risk for disease.

### 2.2. Qualitative Assessment

Each article was examined by two of the reviewers to identify key elements of each study. A first reviewer identified the key elements, which were then confirmed by a second reviewer. Any discrepancies were discussed until consensus was met with 100% agreement. These elements included (1) study design and identification of the methods employed; (2) characteristics of the *participant population*, including overall size (*N*) and demographics information; (3) *measurements* used to assess the PM materials, including comprehension metrics (how well participants retained information from the materials), understandability (how readable and understandable the materials were), trust (participant trust of PM results), preference (what aspects participants liked or did not like about the PM materials), and psychological reactions (emotional responses to the PM materials); (4) *type of PM materials* being assessed; and (5) *type of PM results* presented to the participants, whether hypothetical or genuine patient results. When examining the participant demographics, we also assessed whether health literacy and numeracy were included. Health literacy is defined as one's ability to comprehend and utilize the information regarding healthcare that is needed to make appropriate decisions [18], and numeracy is a measure of one's skills with math concepts and their applications [19]. Articles with similar study designs were identified, and we assessed the similarities and differences within each group of articles. Finally, we compared across groups for overarching themes and takeaways.

## 3. Results and Discussion

**Figure 2:**
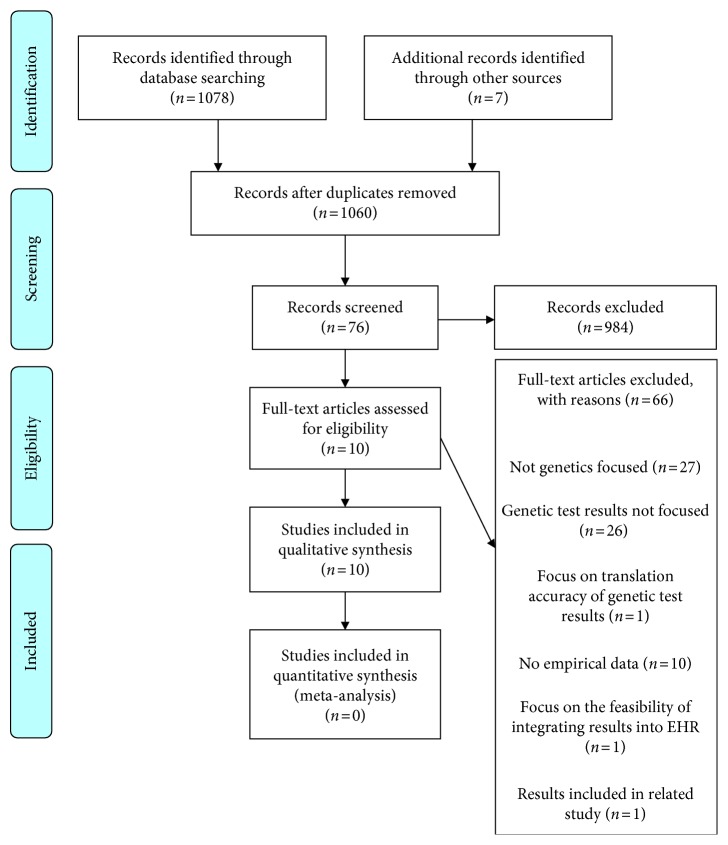
PRISMA 2009 flow diagram illustrating the number of articles included and excluded at each step.

The initial database search resulted in 1078 articles and seven articles was identified by examining the references of relevant articles, totaling 1085 articles. Duplicates were removed, resulting in 1060 article titles, and abstracts were screened for further review. The selection process ultimately resulted in ten full-text articles deemed eligible for inclusion (Figure 2) [17, 18, 20–27].

We assessed methodology, sample size (*N*), and characteristics of the participant population and the PM materials assessed for each article (Table 1).

### 3.1. Individual Study Results

The included articles are summarized and reviewed below with emphasis on the methods used, the study populations, the test report results that were examined, and the differences in the measures used. Three main groups of articles were identified: focus groups and interviews [20, 21, 27] which tested patient-facing PM materials with individuals or small groups of participants and solicited feedback; observational studies [16, 19, 22, 26] which used questionnaires to probe effectiveness of patient-facing PM materials; and randomized control trials [18, 23, 24] which tested patient-facing PM materials by presenting randomized groups with different versions to see which were more effective.

#### 3.1.1. Focus Groups and Interviews

Barajas et al. [20] developed a one-page pictograph-based patient education tool describing the impact of pharmacogenomics on warfarin dosing (an oral anticoagulant, used for the treatment of prevention of thromboembolic disorders). After several iterations of tool development by an interdisciplinary team, the tool was presented to patient focus groups to elicit feedback on content and style via a semistructured interview with open-end questions. These focus groups consisted of five to seven patients already taking warfarin who were recruited at an anticoagulation clinic. More than half of participants (69%) had completed community college or higher levels of education. The study proved a successful pilot of the patient education materials, with positive responses supporting further testing of this tool. These findings also emphasize the importance of involving patient perspectives in the development process of patient education materials. Patient feedback included the following:

- Mixed feelings regarding the incorporation of pharmacogenetics concepts in the handout, some expressing that they did not think the background information was necessary or useful
- Mixed reactions to the use of pictures
- Openness to genetic testing to circumvent the dose adjustment period of warfarin
- Consensus that a handout would be most helpful to patients just starting their warfarin treatment

Stuckey et al. [21] reported on a thematic analysis of parent feedback, collected via interviews and focus groups, regarding the content and design of a novel method of displaying child genomic results in the form of a *Family Report*. Four sets of parents of children with intellectual disabilities were recruited from the participants of a whole genome sequencing study; participation included semistructured interviews and/or focus groups. The *Family Report* was generated based on a provider report, with modifications to promote health literacy and to align with other patient education material studies. In response to participant feedback, the report included a “Next Steps” section, detailed below. The report was accompanied by concept sheets that provided disease-specific information; four designs of this material were also evaluated by the focus groups. Participant feedback included the following:

(v) Preference for a detailed, thorough report (with an initial primary results section) despite the additional length required to convey this content
(vi) Appreciation for
  (a) simple language
  (b) visual aids
  (c) logical (linear) organization of the test results
  (d) glossary for medical terms
  (e) inclusion of “Next Steps” information (list of general topics to discuss with a physician, a list of resources, contact numbers, and support groups)

Liang et al. [27] conducted a qualitative study, using semistructured interviews to assess patient experience with and preference for screening of somatic tumor mutations and their preference for presentation and communication of test results. Sixteen patients with nonsmall cell lung cancer and eight patients with melanoma who had received molecular screening and results were recruited through their medical oncologists at two hospital sites in Sydney, Australia. The mean age of the study participants was 70 years, and the majority were male (58%), married (63%), and not college educated (67%). Health literacy and numeracy were not assessed in this study. The authors found that most study participants understood the need and role of somatic tumor screening, could explain the molecular screening process, and could recall their molecular testing results. Many study participants (46%) expressed that they struggled to retain most of the information from their test results because of information overload and cancer-related distress and thus preferred the following to help with understandability and retention:

(vii) Verbal presentation of test results by their oncologist
(viii) Provision of handouts and other take-home resources to supplement information
(ix) Use of simple, jargon-free language to communicate test results
(x) Exclusion of technicalities and details of tumor screening from test results
(xi) Inclusion of practical matters more relevant to their cancer treatment on test results

While these three studies looked at patient-facing versions of contrasting materials, with one study, Barajas et al. [20] examining educational materials and two studies and Stuckey et al. [21] and Liang et al. [27] examining results and the feedback received during these focus groups and interviews shared common themes. All sets of participants expressed that using simplified language helped them understand the content, and Barajas et al. [20] and Stuckey et al. [21] showed that if designed appropriately, visual aids could be of assistance as well. Participants in pharmacogenomics study (Barajas et al. [20]) expressed mixed feelings towards the visual aids, pointing out that they could add confusion if not designed well. Parents examining the *Family Report* [21] also emphasized the need to make the visual aids appropriate, with concerns about the style of the visual aid not fitting the document and detracting from it. Participants in active cancer treatment with access to an oncologist [27] expressed the need to have take-home materials, presented in simple language, and to supplement information received from their oncologist. These findings suggest that creating patient-facing materials that are written with health literacy levels in mind, with simplified language and glossaries available, can help improve patient engagement. In contrast to the other two studies, Liang et al. [27] relied on retrospective self-reporting by patients, leaving it open to recall bias.

#### 3.1.2. Observational Studies

Leighton et al. [16] compared the comprehension and understandability of hypothetical genetic test results between the general public and genetic counselors. General population participants were recruited via Facebook, a social networking platform, by sending a request for participation to the principal investigator's connections on the site and by posting the request in a variety of groups. The comparison group of genetic counselors was recruited using an email Listserv associated with the National Society of Genetic Counselors. The majority of survey respondents was young, white, and had attained a high level of education. The test result reports used were developed with language pulled from commercially accessible direct-to-consumer (DTC) companies. Both sets of respondents were provided four hypothetical scenarios and asked Likert-scale questions about each scenario that captured their perceived level of genetic risk, level of concern about the results, and perceived understandability of the results. Though the general public participants generally correctly interpreted their results, there was a significant difference in interpretation accuracy and implication of result when compared to the genetic counselors; the general population had significantly fewer correct interpretations than genetic counselors and significantly higher estimation of the usefulness of the results. Health literacy and numeracy were not measured.

Ostergen et al. [22] assessed comprehension of hypothetical genomic testing results by current consumers of commercially available DTC genetic tests. Of note, only current customers were recruited, limiting the study sample to those individuals who can afford DTC genetic testing. Only 15% of participants were nonwhite, 21% had not earned a college degree or higher level of education, and 27% were aged greater than 60 years. Numeracy and genetic literacy were assessed prior to receiving genetic test results; both characteristics were high in this patient population (an average score of 4.7 out of 5 for numeracy and average score of 8.15 out of 9 for genetic literacy). The results indicated generally high comprehension of test results, with lower performance for scenarios about phenylketonuria and cystic fibrosis, diseases requiring an understanding of recessive traits. Participant numeracy, genetic knowledge, education level, race, and age had significant effects on comprehension. The authors concluded that genetic test results need to be tailored to the consumer characteristics and to the test type in order to maximize understanding.

Kaphingst et al. [19] conducted a three-part survey with members of a health maintenance group to collect data on comprehension, recall, and interpretation of genetic test results. Middle-aged (25–40) participants were recruited from a health maintenance organization, and underrepresented populations were intentionally oversampled, resulting in a sample that was 43% male, 62% white, and 52% college educated. The authors created their own genetic report to provide to participants; language was plain, jargon was limited, content was limited to essential information, information was summarized, and key information was highlighted. The study reported a high success rate of information recall and limited deterministic interpretation of the genetic test results. The authors found that a more deterministic interpretation was associated with confusion by the presented test results, lower educational attainment, and being of racial and ethnic minority groups. Health literacy and genetic self-efficacy, though collected, were not reported as statistically significant covariates.

Olson et al. [26] assessed comprehension, understandability, and attitudes towards pharmacogenomic test results. Participants were recruited from the Mayo Clinic Biobank to undergo genetic testing to determine their CYP2D6 (an enzyme involved in the metabolism of up to 25% of all prescribed medication) metabolizer phenotype. Following genetic testing, a letter summarizing their pharmacogenomic test results, two pages of educational materials about pharmacogenomic testing, instructions on how to log on to the patient portal to view their full test report, and a survey were mailed to the study participants. On the survey, participants were asked Likert-scale questions about the understandability of their results, as well as their comprehension and ability to explain their pharmacogenomic test results. The survey also included open-end questions, asking them to provide recommendations on how to best improve the pharmacogenomic test results summary letter. A majority of the 869 study participants were white (98%), 55% were female, 57% had four or more years of postsecondary education, and the mean age was 59 years. Health literacy and numeracy were not assessed in this study. The authors found that about one-third of the respondents (33%) did not understand their pharmacogenomic test results and that more study participants understood the pharmacogenomic test results summary letter (67%) than understood the full test results as displayed in the patient portal (53%). In addition, survey respondents with a higher level of education were more likely to understand their genetic test results and showed more confidence in their ability to explain their genetic test results to someone else. Several themes emerged from the respondents' suggestions to improve the pharmacogenomic test results summary letter, including the following:

(xii) Use of plain and simplified language on the summary letter
(xiii) Having someone deliver and explain the results to them in person or over the phone
(xiv) Personalizing the results to make it relevant to individual patient's care
(xv) Simplifying the layout and content of the pharmacogenomic test results by including tables and graphs

Three of the observational studies captured in this systematic review assessed consumer's understanding of direct-to-consumer genetic testing results. Leighton et al. [16] as well as Ostergen et al. [22] used commercially available genetic testing results, while Kaphingst et al. [19] designed their own report that was sent directly to consumers. Only one study (Olson et al. [26]) assessed patient understanding of genetic testing results by having patients undergo pharmacogenomic testing and providing them with the results. All studies showed a high level of understanding, but when examined closely, this finding does not suggest that the general population has high understanding of genetic testing results. Leighton et al. [16], Ostergen et al. [22], and Olson et al. [26] had biased samples, with Leighton et al. [16] using the principal investigator's connections on a social media networking site and Ostergen et al. [22] only using patients who were already enrolled in DTC genetic testing. Olson et al. [26] only sampled patients in the Mayo Clinic Biobank and thus are likely more informed about genetic testing. As such, these participant groups are unlikely to represent the general population of the United States. Kaphingst et al. [19] worked to oversample underrepresented populations and had a more representative sample but did not use language from commercially available DTC results. Instead, these authors, with the exception of Olson et al. [26], designed their own results, using language that could accommodate a wider range of health literacy levels. These results, taken together with recommendations from patients in the Olson et al. [26] study, can suggest that high comprehension is possible when effort is made to present the information in a digestible manner.

#### 3.1.3. Randomized Controlled Trials

Brewer et al. [23] presented hypothetical recurrence risk test results in six format variations to a cohort of patients with an early stage breast cancer diagnosis. Accuracy of recall and attitudes (e.g., confidence and trust) towards test results were assessed. Each risk format included an additional graphic or piece of information, such as continuum graphics, confidence intervals, risk graphs, assay descriptions, and icon arrays. Results generally indicated that the simplest graphic, which displayed risk as a percentage and categorized it as low, moderate, or high, was the best communication format in terms of accuracy of recall and participant preference. Additionally, the study found that high- and low-risk results were easier to understand than moderate risk. Health literacy was not statistically significant, though higher numeracy was associated with higher accuracy, understanding, and trust of test results. Of note, the standard format was used to communicate results to the patients, and this format was associated with the most errors and was the most disliked format.

Giuse et al. [18] investigated the impact of utilizing simplified genetic language and different learning modalities on comprehension and understandability of a decision support tool. These authors adapted publicly available genetic decision support content designed for providers and tested three versions using a cohort of patients or caregivers of patients with melanoma. The first version was the original website, geared towards researchers and clinicians. The second version was the original text, but with links to short explanatory videos that described the more complex genetic terms. The third version was translated to a sixth-grade reading level and also incorporated short explanatory videos. Understandability of and satisfaction with the information were assessed using a survey, and comprehension was assessed using before and after knowledge questionnaires. The authors concluded that consumer-level translated results are the most beneficial to performance, though the short knowledge videos facilitate learning when combined with the professional-level information. Age was correlated with comprehension, and while health literacy was captured, the majority of the participants had adequate health literacy, as measured by a subjective health literacy assessment. This lack of variation in health literacy made it hard to draw conclusions on how this tool may help patients with low health literacy.

Shaer et al. [24] conducted a three-pronged study to develop interactive visualizations of genomic data and test their effectiveness at facilitating comprehension. The majority of participants were college educated (83%); race, age, health literacy, genetics knowledge, and numeracy were not assessed. A needs assessment with early adopters of genetic testing services was conducted followed by a qualitative analysis of interviews seeking to understand how current users engage with their genetic test results. Finally, the authors randomly assigned one of the developed visualizations and tested participant comprehension using Amazon Mechanical Turk to recruit the participants. Conclusions favor the use of visual aids and visual representation of data to improve comprehension.

The three randomized controlled trials captured by this systematic review assessed varying types of patient-facing PM materials using experimental rigor to test how aspects of the design influence patient understanding. All demonstrated that using visual aids, such as videos and data visualizations, increases patient understanding. Another key takeaway is that simplicity of the content also helps increase comprehension; when translated to a lower reading level, patients had improved comprehension (Giuse et al. [18]) and eliminating unnecessary interaction with the display enhanced understanding (Shaer et al. [24]).

### 3.2. Comparisons across Studies

In the following section, characteristics of the studies, including study population, measured outcomes, and education materials, are compared to provide further insight to how this group of studies can inform the creation of patient-facing PM materials and what work remains to be done.

#### 3.2.1. Participant Population

Of the nine studies that reported demographic data, none sought to focus on populations that have been identified in the literature as having typically lower health literacy and numeracy levels [16, 19, 20, 22–24, 26, 27]. While not specifically targeting individuals with low numeracy or health literacy, one study did attempt to oversample patient populations typically underrepresented in this type of research—male, not college educated, and African American; despite these efforts, the majority of participants were still college educated and white [19]. The majority of participants in all of the studies were white and educated, with the exception of one study, Liang et al. [27], where the majority of the participants were not college educated, and though several studies reported results of a correlation between higher health literacy or numeracy, the average health literacy and numeracy of those study populations was relatively high [18, 23]. Most studies identified this as a limitation or addressed in their discussion [16, 19, 20, 22–24, 26, 27]. Future work should ensure that study designs seeking to improve patient-facing PM materials include individuals with low levels of health literacy. Additionally, several participant populations had previous experience with genetic test results or high genetic knowledge [21–24, 26, 27], though one study reported no significant correlation between individuals with prior genetic testing experience and those without [23].

#### 3.2.2. Measurement Definitions

All of the randomized controlled trials and survey studies analyzed participant comprehension of genetic test results as their primary measure [16, 19–24, 26, 27]. This was collected through knowledge questionnaires [18, 22, 24, 26, 27], and errors related to recall of the information were included in the genetic test results [19, 23]. One study defined comprehension as a comparison of the participant's perceived level of genetic risk compared to that of a group of genetic experts [16]. A breakdown of how these metrics were quantified can be found in Table 2. Five studies collected participant preference of content and style for genetic result reports or patient education materials [18, 20, 21, 23, 26, 27].

#### 3.2.3. Genomic Test Report

The content and organization of the genomic test results were the primary focus of eight of the ten studies [16, 19, 21–27]. These eight studies utilized multiple research methodologies to investigate the impact of the content, including both qualitative analysis of focus groups [21], semistructured interviews [19, 21, 27], or open-end survey response and quantitative knowledge tests [16, 19, 22, 26, 27]. One of the studies developed their own content based on the health literacy literature but did not compare study measures with a control group or analyze feedback on the new content [19]. It is important to note that several of these studies used hypothetical results, not actual results [16, 22, 23]. The hypothetical aspect of these reports may influence the way participants respond to them such that there may be less engagement with hypothetical results than with actual results. However, these studies still provide insight to what can aid comprehension and the use of hypothetical results may enable studies to extend the sample beyond individuals who have the means or interest to participate in genetic testing. Aspects of the developed materials that were found to aid comprehension of genetic test results include visual aids and simplified language. The remaining two included articles assessed patient-facing PM educational tools, as opposed to results. These studies also identified visual aids and simplified language as factors that enhance patient's understanding of PM [18, 20].

### 3.3. Limitations

The conclusions of our review are limited by the small number of papers we were able to identify on the topic of patient education and patient-facing genetic results reports in PM. Similarly, comparison of the findings was limited by the various methodologies utilized across the studies; focus groups and interviews have different results than randomized control trials or observational studies. While we did not conduct a formal bias assessment of the articles reviewed, we did address their limitations within each article summary to acknowledge any shortcomings in generalizability of results and opportunities for future study. This review is also limited by the decision to use broad key terms such as “precision medicine” and not gene specific key words such as BRCA, which may have resulted in studies using those key words to be missed.

## 4. Conclusions

Healthcare systems are gradually moving towards new models of care based on integrated care processes shared by different care providers and on an empowered role of the patient. As PM gains traction, there is going to be higher demand for patients to understand how it influences their care. From primary care to oncology, PM is increasing, and a wider population of patients will be required to interact with PM results. The studies described provide important groundwork by highlighting consistent aspects of design that aid in comprehension, including appropriately designed visual aids and simplified language. Through our systematic review of the literature, it is evidenced that the breadth of knowledge in this field is limited in scope, given the participant populations. Evidence shows that a patient's health literacy impacts their ability to understand PM materials, making it essential that the patient-facing materials are tested across a wide variety of health literacy levels. PM offers great potential to tailor care to the individual, but for this potential to be reached, patients must be included in the process.

## Figures and Tables

**Table 1:** Brief summary of the methodology, sample size (*N*), and characteristics of participant population, PM materials, and PM results type used in the included articles.

Citation	Methodology	*N*	Participant population characteristics	PM materials assessed	PM results type
Barajas et al. [20]	Focus groups	17	Patients currently taking warfarin	Educational tool developed as part of the study design	Hypothetical scenarios
Brewer et al. [23]	Randomized trial	133	Patients with positive early stage breast cancer diagnosis	Results developed as part of the study design	Hypothetical results
Giuse et al. [18]	Randomized controlled trial	88	Patients with positive melanoma diagnosis or their caregivers	Educational tools developed as part of the study design	Hypothetical results
Kaphingst et al. [19]	Observational	199	Patients enrolled in health maintenance organization	Results developed from best practices in health literacy	Actual patient results
Leighton et al. [16]	Observational	145	General public	Commercially available DTC report	Hypothetical results
		171	Genetic counselors		
Liang et al. [27]	Interview	16	Patients with nonsmall cell lung cancer	Results previously provided from molecular testing	Actual patient results
		8	Patients with melanoma		
Olson et al. [26]	Observational	869	Patients in the Mayo Clinic Biobank	A summary and full report of pharmacogenomic test results	Actual patient results
Ostergen et al. [22]	Observational	1030	Current consumers of personal genomic testing	Commercially available DTC report	Hypothetical results
Shaer et al. [24]	Randomized controlled trial	730	General public	Results developed as part of the study design	Hypothetical results
Stuckey et al. [21]	Semistructured interviews; focus groups	9	Parents of children enrolled in genome sequencing clinical research study	Results developed as part of the study design	Hypothetical results

**Table 2:** Measurement definitions.

	Knowledge metrics	Understandability	Trust	Preference	Psychological reactions
Barajas et al. [20]	—	Discussion about the content of the tool	—	Discussion about the content of the tool	Discussion about openness to testing

Brewer et al. [23]	1. Gist recall: low/med/high risk 2. Verbatim recall: risk percentage	1. “How confident are you that you understand this test result?” 2. “How easy was this test result to understand?”	“How much do you trust that this test result is accurate?”	Rank 6 formats from liked most to liked least	—

Giuse et al. [18]	Before and after 10-question knowledge quiz about genetics, the disease, and the mutation		—	—	—

Kaphingst et al. [19]	1. Free recall 2. Prompted recall	Likert-scale ratings of the deterministic nature of results	Likert-scale ratings of the believability, reliability, completeness, helpfulness, difficulty, and accuracy of the information	—	Adapted Positive and Negative Affect Scale [25]

Leighton et al. [16]	Specifics unavailable, compared perceived risk between general public and genetic counselors	Specifics unavailable	Belief that results would be helpful in deciding future medical management	—	Level of concern

Liang et al. [27]	Free recall	Discussion about understanding of somatic tumor screening	Discussion about views, perceived advantages and disadvantages of somatic tumor screening	Discussion about information and communication preferences	Discussion about psychological support needs

Olson et al. [26]	Questionnaire	Questionnaire	Likert-scale ratings of participant attitudes and usefulness of pharmacogenomics testing	Open-end questions about improvement of results letter	Question about opening to encourage others to get pharmacogenomic testing

Ostergen et al. [22]	Accuracy scores	Likert-scale ratings of the deterministic nature of results	Likert-scale ratings of the believability, reliability, completeness, helpfulness, difficulty, and accuracy of the information	—	—

Shaer et al. [24]	Questionnaire	Questionnaire	—	Questionnaire	

Stuckey et al. [21]	—	Discussion and interview questions about the content	—	Discussion about content	Discussion about desire for Next Steps
